# Spatial pattern of intra-laminar connectivity in supragranular mouse auditory cortex

**DOI:** 10.3389/fncir.2014.00015

**Published:** 2014-03-11

**Authors:** Paul V. Watkins, Joseph P. Y. Kao, Patrick O. Kanold

**Affiliations:** ^1^Department of Biology, University of Maryland, College ParkMD, USA; ^2^Center for Biomedical Engineering and Technology, University of Maryland School of Medicine, BaltimoreMD, USA; ^3^Department of Physiology, University of Maryland School of Medicine, BaltimoreMD, USA; ^4^Institute for Systems Research, University of Maryland, College ParkMD, USA

**Keywords:** auditory cortex, microcircuit, intracortical, excitation, inhibition, mouse

## Abstract

Neuronal responses and topographic organization of feature selectivity in the cerebral cortex are shaped by ascending inputs and by intracortical connectivity. The mammalian primary auditory cortex has a tonotopic arrangement at large spatial scales (greater than 300 microns). This large-scale architecture breaks down in supragranular layers at smaller scales (around 300 microns), where nearby frequency and sound level tuning properties can be quite heterogeneous. Since layer 4 has a more homogeneous architecture, the heterogeneity in supragranular layers might be caused by heterogeneous ascending input or via heterogeneous intralaminar connections. Here we measure the functional 2-dimensional spatial connectivity pattern of the supragranular auditory cortex on micro-column scales. In general connection probability decreases with radial distance from each neuron, but the decrease is steeper in the isofrequency axis leading to an anisotropic distribution of connection probability with respect to the tonotopic axis. In addition to this radial decrease in connection probability we find a patchy organization of inhibitory and excitatory synaptic inputs that is also anisotropic with respect to the tonotopic axis. These periodicities are at spatial scales of ~100 and ~300 μm. While these spatial periodicities show anisotropy in auditory cortex, they are isotropic in visual cortex, indicating region specific differences in intralaminar connections. Together our results show that layer 2/3 neurons in auditory cortex show specific spatial intralaminar connectivity despite the overtly heterogeneous tuning properties.

## INTRODUCTION

The ability of many mammalian species to analyze auditory scenes requires an exquisite representation of the auditory world. The primary auditory cortex (A1) is a key structure underlying the perception of sounds and the processing of auditory information. On a large scale the main organizational principle of A1 is the tonotopic axis (Merzenich et al., 1973, 1975), which is inherited from the 1-dimensional sensory epithelium of the cochlea and also present at lower levels of the auditory system. In addition, A1 contains patchy organization of other sound features within isofrequency bands, possibly indicating functional specializations of A1 (Reale et al., 1983; Matsubara and Phillips, 1988; Read et al., 2001; Ojima and Takayanagi, 2004; Ojima et al., 2005). In contrast to the smooth maps of frequency preference obtained with low-resolution techniques, recent *in vivo* 2-photon imaging studies in mice have revealed that the frequency organization of neurons in supragranular layers (layer 2/3) on a local scale is very heterogeneous (Bandyopadhyay et al., 2010; Rothschild et al., 2010). Since similar studies revealed that the frequency organization in layer 4 is more homogeneous than in layer 2/3 (Guo et al., 2012; Winkowski and Kanold, 2013), this raises the question of which microcircuits underlie the differences in frequency preference of nearby neurons within and between layers.

Layer 2/3 of A1 is known to contain many cortico-cortical connections (Song et al., 2006), thus it might contain specific connectivity patterns at small spatial scales, referred to as microcircuitry, that could underlie the observed heterogeneity in frequency preference of nearby neurons. Prior anatomical studies in cat have shown spatially biased inhibitory projections on large spatial scales (Yuan et al., 2011a,b). *In vitro* studies using paired recordings in thalamocortical slices from mice have indicated a general radial decrease in intralaminar connection probability for excitatory and inhibitory connections (Oswald et al., 2009). *In vitro* photostimulation experiments using slices that preserve either the tonotopic or the isofrequency axis have indicated a spatial anisotropy of excitatory connections in inputs from infragranular layers (Oviedo et al., 2010). Here we directly investigate if excitatory and inhibitory connection probability and connection strength in A1 layer 2/3 show anisotropies and also determine if the spatial anisotropies of intracortical connections are a unique feature of A1 or a general characteristic of sensory cortex.

To investigate the existence and spatial arrangement of excitatory and inhibitory microcircuits in layer 2/3 of mouse auditory cortex we developed a tangential slice preparation in which intralaminar connections are preserved in all spatial directions. We find that layer 2/3 of auditory cortex contains excitatory and inhibitory microcircuits that on average demonstrate a decrease in connection probability as a function of the distance from each cell-center. We also find that connection probability and connection strength shows spatial patchiness and that the spatial patchiness is anisotropic relative to the tonotopic axis. However, we find that the visual cortex lacks the anisotropy of this patchy arrangement of synaptic connections. Together these results indicate the presence of an intermingled connectivity pattern in auditory cortex that shapes its responses. Furthermore, our results indicate that the cerebral cortex is not a uniform processor, but that different sensory regions contain microcircuits that are specialized for a particular processing task.

## MATERIALS AND METHODS

All procedures followed the University of Maryland College Park animal use regulations. *In vivo* and *in vitro* physiology methods are similar to our prior studies previously (Zhao et al., 2009; Viswanathan et al., 2012).

### SLICE PREPARATION

**FIGURE 1:**
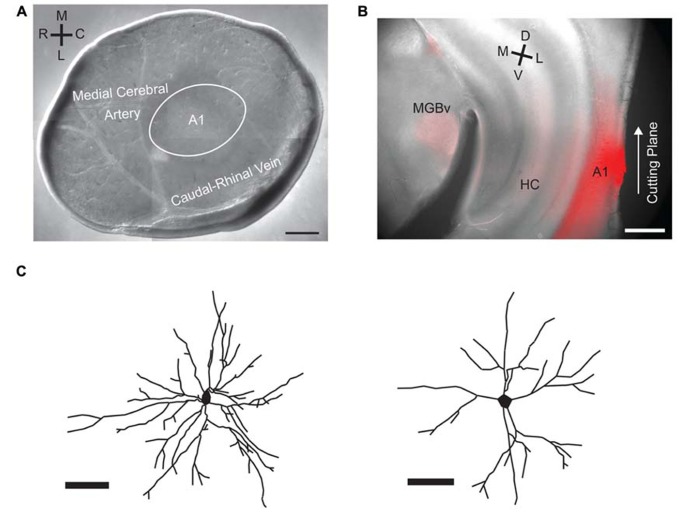
**A1 tangential slice preparation and biocytin stains of basal dendritic arbors.** **(A)** DIC image of the A1 tangential slice with the vasculature preserved. Labels indicate approximate position of A1 relative to the vasculature. **(B)** Retrograde labeling of auditory thalamus (MGN) after insertion of DiI into remaining brain block after A1 tangential slice has been cut. **(C)** Manual reconstructions of basal dendritic arbors from sample cells labeled with biocytin contained in recording pipette. Scale bars 500 μm in **A**, **B**, 50 μm in **C**.

Mice (C57BL/6) of either sex between P15–P25 were deeply anesthetized with isofluorane (Halocarbon). A block of brain containing A1 and the medial geniculate nucleus (MGN) was removed and tangential slices (400 μm thick) were cut on a vibrating microtome (Leica) in ice-cold ACSF containing (in mM): 130 NaCl, 3 KCl, 1.25 KH_2_PO_4_, 20 NaHCO_3_, 10 glucose, 1.3 MgSO_4_, 2.5 CaCl_2_ (pH 7.35–7.4, in 95% O_2_–5% CO_2_). Slices were cut by blocking the brain in a direction perpendicular to the thalamocortical cutting angle of 15° (Zhao et al., 2009) and then removing approximately 150 μm from the tangential surface. Before slicing, a cut was made in medial-lateral (ML) direction of the intact brain, at the rostral end of the approximate slice location. This straight cut was used so the slice could be oriented in the recording chamber with respect to the ML and rostral-caudal (RC) directions. The recording location in A1 was confirmed by comparing with the location of intact vasculature on the tangential slice (**Figure 1A**) and inserting DiI into the remaining hemisphere and confirming retrograde labeling of MBGv (**Figure 1B**). Tangential visual slices were created by blocking orthogonal to the thalamocortical visual slice angle (MacLean et al., 2006) for a horizontal cutting angle 35° from rostral and a coronal angle 65° relative to the hemispheric midline. Slices were incubated for 1 hour in ACSF at 30°C and then kept at room temperature. For recording, slices were held in a chamber on a fixed stage microscope (Olympus BX51) and superfused (2–4 ml/min) with ACSF at room temperature to reduce spontaneous activity in the slice. 50 μM (2R)-amino-5-phosphonovaleric acid (AP5, NMDA antagonist) was added to reduce excitability for photostimulation experiments performed at 40x magnification.

### ELECTROPHYSIOLOGY AND PHOTOSTIMULATION

Whole-cell recordings were performed with a patch clamp amplifier (Multiclamp 700B, Axon Instruments, CA, USA) using pipettes with input resistance of 4–8 MΩ. Data acquisition was performed by National Instruments AD boards and custom software (Ephus; Suter et al., 2010). Ephus (http://www.ephus.org) is written in MATLAB (Mathworks, MA, USA) and was adapted to our setup. Voltages were corrected for an estimated junction potential of 10 mV. Electrodes were filled with (in mM) 115 cesium methanesulfonate (CsCH_3_SO_3_), 5 NaF, 10 EGTA, 10 HEPES, 15 CsCl, 3.5 MgATP, 3 QX-314 (pH 7.25, 300 mOsm). Biocytin or Neurobiotin (0.5%) was added to the electrode solution as needed. Series resistances were typically 20–25 MΩ. 

#### Photostimulation

0.5–1 mM caged glutamate (*N*-(6-nitro-7-coumarylmethyl)-L-glutamate; Ncm-Glu; Kao, 2006) was added to the ACSF. Without UV light, this compound has no effect on neuronal activity (Kao, 2006). UV laser light (500 mW, 355 nm, 100 kHz repetition rate, DPSS, Santa Clara, CA, USA; 1–2 ms pulses) was split by a 33% beam splitter (CVI Melles Griot), attenuated by a Pockels cell (Conoptics), shuttered (NM Laser), and coupled into a microscope via scan mirrors (Cambridge Technology) and a dichroic mirror (Shepherd et al., 2003). The laser beam entered the slice axially through an objective (Olympus 40×, 0.80 NA/water or Olympus 10×, 0.30 NA/water) and had a diameter of <20 μm at 10× and <8 μm at 40×. Laser power at the sample was 15–25 mW. We varied laser power and determined that this power provided reliable activation of neurons (Viswanathan et al., 2012). For high magnification maps (40× objective) we stimulated in a 25 × 25 grid with locations spaced 8 μm apart, enabling us to probe areas of <200 μm per side For lower magnification maps (10× objective) we stimulated in a 30 × 30 grid with locations spaced 30 μm apart, enabling us to probe areas of <800 μm per side. Photostimuli were applied at 1 Hz.

#### Analysis and statistics

Calculation of PSC magnitudes based on recorded responses at different photostimulation locations was performed essentially as described previously (Shepherd et al., 2003; Zhao et al., 2009; Viswanathan et al., 2012) with custom software written in MATLAB. To detect monosynaptically evoked post-synaptic currents (PSCs) we detected peak PSC amplitudes in a ~100ms time window after the stimulation. We measured both peak amplitude and transferred charge. Transferred charge was measured by integrating the PSC. Traces containing a short-latency (<8 ms) response were used as direct currents for high magnification maps. The latency cutoff of 8 ms was utilized because this was a good divider for separating out the majority of very large amplitude inward currents, which were direct currents. This latency cutoff also did not exclude any outward currents that were not extremely small, except for a very small number of low latency outward currents (Brill and Huguenard, 2009).

For the population of recorded neurons we aligned maps by centering them with the recorded soma at the origin. The mapping coordinates did not need to be rotated in order to be aligned with the ML and RC directions because the straight cut on the rostral end of the slice (see above) was always aligned in the same orientation in the recording chamber. Maps either plotted the probability of obtaining a PSC (>8 ms latency) at each stimulus location, which we refer to as the connection probability maps, or the average transferred charge of the PSCs at each stimulus location, which we refer to as mean strength maps. We measured connection probability as the total number of times an event occurred at a photostimulation location divided by the total number of times that location was photostimulated over all recorded neurons. We measured mean strength as the mean transferred charge for events that occurred at a photostimulation location. The mean transferred charge was first normalized to range between 0 and 1 for each recorded neuron map individually and then normalized maps were averaged over the population of recorded neurons. We also calculated connection probability and mean strength averaged across one of the cardinal directions (RC or ML) which we refer to as marginal profiles, or averaged in radial distance or angle from the soma (polar coordinates), which we refer to as radial and angular profiles, respectively.

Statistics were utilized to compute confidence intervals (CIs) for connection strength maps, mean strength maps, marginal profiles, radial and angular profiles and spatial frequency maps (2D Fourier Transforms, see below). CIs were generated using a bootstrap technique (Efron and Tibshirani, 1994). The bootstrap was performed by randomly resampling responses at each photostimulation location. Chosen responses were replaced into the pool being randomly resampled after each selection so they could potentially be sampled again on subsequent resamplings (bootstrapping). The resampling was performed 1000 times and the 2.5 and 97.5 percentiles of the responses (either mean strength, connection probability or magnitude of spatial frequency) over the 1000 repeats were used as the top and bottom of the 95% CI. We measured significance of these responses by comparing them to “shuffled maps.” Shuffled maps were created by randomly re-assigning each response to a different photostimulation location. This re-assignment was performed without replacement such that each location re-assignment was a random permutation of the original ordering of photostimulation locations (permutation test). This procedure creates a shuffled version of the responses but with the same set of photostimulation locations. The resulting maps (or marginal profiles) typically have a flat spatial structure and thereby give an estimate of what the response map would look like if responses were uniformly distributed across all photostimulation locations (the null hypothesis). Shuffled responses were subject to the same bootstrap test as for the unshuffled responses in order to calculate the 95% CI for the null hypothesis of spatially- uniform responses.

Spatial frequency maps were calculated with 95% CIs in the same manner as described for the mean response maps except that a 2D Fast Fourier Transform (FFT) as implemented in the Matlab algorithm was calculated on both unshuffled and shuffled response maps prior to the bootstrap test. The 2D FFT was applied after detrending and demeaning by removing the best-fit plane from the response maps.

Photostimulation locations in each response map or marginal profile were significant (bootstrap test on shuffled and unshuffled responses, see above) if the upper confidence interval of the shuffled responses was greater than the lower confidence interval for the unshuffled responses. This significance level was at *p* < 0.05 because 95% CIs were compared.

#### Drugs

We used TTX (1 μM) to block action potentials. All chemicals and drugs were obtained from Sigma.

## RESULTS

To investigate if layer 2/3 microcircuits in A1 have a spatial anisotropy, we use a tangential slice preparation in which the full extent of layer 2/3 is kept intact (**Figure 1A**). Since the vasculature can be preserved in the slice (Dorr et al., 2007), the slice can be oriented with respect to the *in vivo* cardinal directions of RC and ML. The RC direction mostly corresponds to the tonotopic axis of auditory cortex in the mouse (Stiebler et al., 1997). We confirm the location of A1 by injecting DiI in the remaining block of brain (not sliced) that contained layer 4 and thalamus and then verifying that the MGN is retrogradely labeled (**Figure 1B**). We record from layer 2/3 neurons with whole-cell patch clamp techniques and perform laser-scanning photostimulation (LSPS; Shepherd et al., 2003; Barbour and Callaway, 2008; Zhao et al., 2009; Viswanathan et al., 2012). To confirm the identity of recorded neurons we reconstruct cell morphologies by including biocytin in the recording pipette. We find that our recordings are obtained from pyramidal cells, which showed extensive basal dendritic arbors (**Figure 1C**).

**FIGURE 2:**
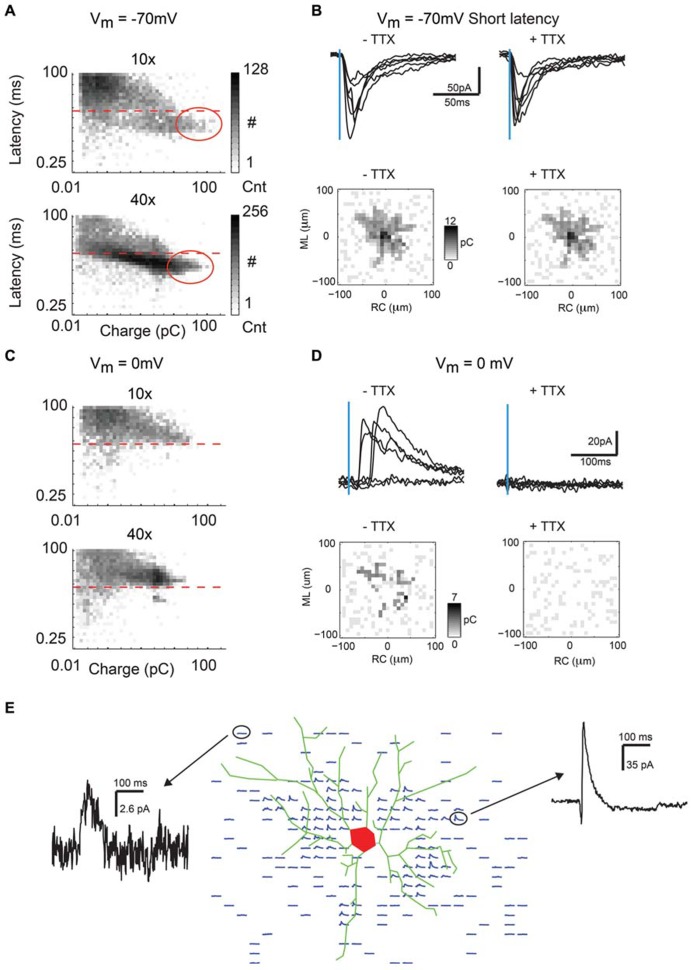
**Bath application of TTX extinguishes IPSC but not direct currents recorded near the soma.** **(A)** Two dimensional histogram (log scale event counts) of latency vs. charge for all inward currents recorded in response to photostimulation in our dataset (*N* = 16250 events for 10×, *N* = 20625 events for 40×). Dashed line indicates the 8 ms cutoff used to distinguish direct from synaptic events. Notice the cutoff mostly separates out the largest and fastest events that are direct currents. The approximate area of the largest direct currents is circled. **(B)** Direct currents are large-amplitude and short-latency inward currents that result from direct excitation of the recorded neuron by glutamate photorelease near the soma or proximal dendrites (top left). These currents are resistant to TTX (top right). Maps (from single neurons shown in top panels) showing total charge of direct events created before and after bath TTX application are very similar (bottom), typically indicating the extent of the neuron’s basal dendrites in the tangential slice preparation. **(C)** Two dimensional histogram (log scale event counts) of latency vs. charge for all outward currents recorded in response to photostimulation in our dataset (*N* = 15000 events for 10×, *N* = 19375 events for 40×). Notice except for a small number of very small amplitude events and a small cluster of fast outward currents, all currents are at a latency of greater than 8 ms indicative of IPSCs. **(D)** Outward currents when holding at 0 mV typically occur at a latency of 8 ms or greater and are IPSCs (top left), as they are blocked by bath application of TTX (top right). Charge maps created before and after bath TTX application (bottom) show that IPSCs are mostly absent when spiking is blocked. **(E)** Spatial map of IPSCs evoked by photostimulation overlaid with the soma and basal dendritic morphology reconstructed from biocytin stain.

Single-photon LSPS using caged glutamate activates glutamate receptors on neurons and is typically used to probe for synaptic connections (Callaway and Katz, 1993; Shepherd et al., 2003; Barbour and Callaway, 2008; Zhao et al., 2009; Viswanathan et al., 2012). Photoactivation of caged glutamate typically evokes two types of responses in the recorded neuron when it is held at resting membrane potential (about -70 mV). The first type of response is characterized by a large inward current with short latency and is due to direct activation of glutamate receptors on the soma and proximal dendrites of the neuron under study (direct response, **Figure 2A** below dashed line). The second type of response is characterized by a smaller amplitude inward current with longer latency. This evoked response, or excitatory post-synaptic current (EPSC), indicates that a connection is present between a stimulated neuron and the recorded neuron. Direct currents are separated out from synaptic currents by using a latency cutoff of 8 ms (**Figure 2A**, dashed line), which demonstrates a good delineation between fast, large amplitude direct currents and synaptic currents when the population of events is plotted as a two-dimensional histogram of charge vs. latency (**Figure 2A**). To test if short latency currents represent synaptic events or direct currents, we map a 200 × 200 μm area around soma. Short latency inward currents remain in the presence of TTX, indicating that they are caused by direct activation of the soma or proximal dendrites (**Figure 2B**, top). The total charge of direct events is calculated at each LSPS location (25 × 25 grid; 8 μm grid spacing) and then is plotted relative to the location of the recorded neuron, creating a map of direct currents. Direct maps recorded before and after application of TTX were practically identical (**Figure 2B**, bottom).

In addition to mapping excitatory connections, the LSPS technique can also be used to map inhibitory connections by holding the recorded neuron at the glutamate reversal potential of 0 mV. At this potential direct and excitatory responses are absent, and only relatively long-latency (>8 ms) and long-lasting outward currents are observed (**Figure 2C**, above dashed line). These outward currents could be blocked by application of TTX and thus are evoked inhibitory post-synaptic currents (IPSCs; **Figure 2D**, top). Maps of IPSC charge show only very small spontaneous currents after application of TTX, indicating that the outward currents are synaptically evoked (**Figure 2D**, bottom). Although IPSCs are observed over much of the map, they are obscured (presumably by shunting) by direct currents for some neurons at locations near the soma of the recorded neuron. IPSCs in a single neuron could be evoked from spatially non-uniform locations in the mapped area. An overlay of the reconstructed neuronal morphology with the spatial map of IPSCs demonstrates that many IPSCs are located in the vicinity of dendrites, but do not necessarily directly overlap them (**Figure 2E**). This suggests that many inhibitory neurons projecting to pyramidal cells are located close to the dendrite, while some inhibitory neurons are located further away.

### INTRALAMINAR CONNECTION PROBABILITIES IN LAYER 2/3 OF A1 ARE ANISOTROPIC AND SHOW ANISOTROPIC PERIODICITIES

**FIGURE 3:**
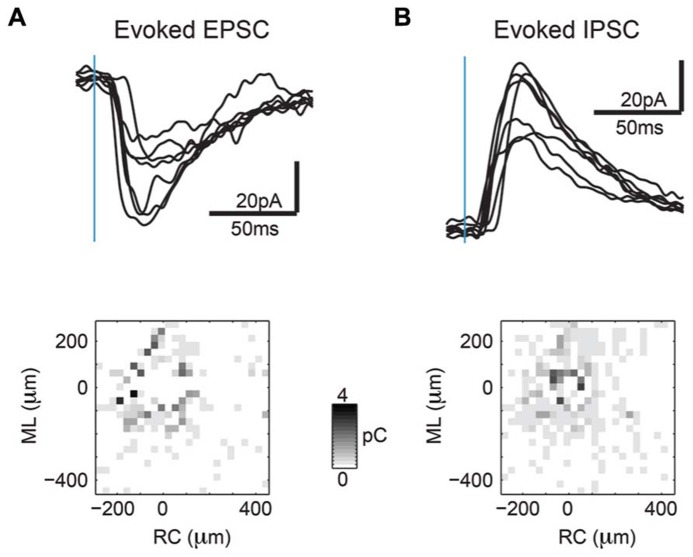
**Sample synaptic current traces from area photostimulated further from the soma show patchy connectivity.** **(A)** Sample inward currents (>8 ms latency) are EPSCs indicating excitatory synaptic connectivity (top). Map of inward current charges shown at different photostimulation locations shows patchiness of excitatory inputs at locations further away from soma (bottom). **(B)** Sample outward currents (>8 ms latency) are IPSCs indicating inhibitory synaptic connectivity (top). Map of outward current charges shown at different photostimulation locations show rounder extent of inhibitory inputs but are also patchy at locations further away from soma (bottom).

In order to investigate connectivity of layer 2/3 neurons in A1 relative to the tonotopic axis, we perform LSPS on auditory tangential slices at low magnification (10× objective, allowing a mapped area of approximately 700 × 700 μm). The high spatial mapping resolution of 30 μm relative to the functional resolution of ~100 μm (Viswanathan et al., 2012; see **Figure 2**) causes activation of a neuron from neighboring spatial locations, and thus represents spatial oversampling. At these distances both EPSCs (**Figure 3A**, top) as well as IPSCs (**Figure 3B**, top) could be mapped. Maps of single neurons at this magnification often show more connectivity near the soma, and spatially non-uniform regions of distant input (**Figures 3A,B**, bottom).

**FIGURE 4:**
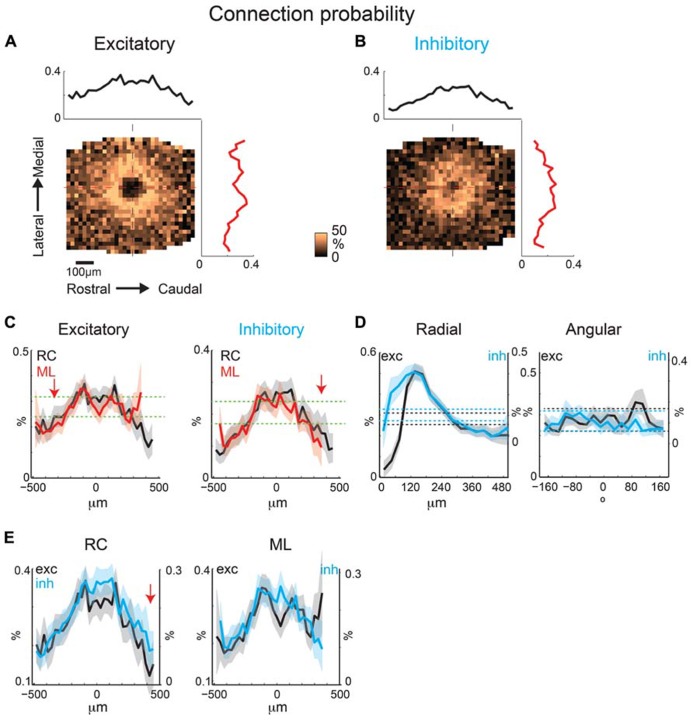
**Mean excitatory and inhibitory connection probability maps from a population of neurons recorded at 10× magnification.** Mean maps from a population of recorded neurons held at resting membrane potential (**A**; *N* = 26) and glutamate reversal potential (**B**; *N* = 24) generally demonstrate the 2D Gaussian connectivity profile. Plots above and to the right of the mean map indicate the marginal profile along the RC (tonotopic) and ML (isofrequency) directions, respectively. **(C)** ML and RC marginal profiles from **(A,B)** overlaid for excitatory (left) and inhibitory (right) inputs. Green dashed lines indicate the 95% confidence interval for the mean of the shuffled image. The shaded areas for this and subsequent plots represent the 95% confidence interval for the marginal profiles. Arrows highlight slightly wider extent in RC direction. **(D)** Mean map polar marginals where radial profile (left) is a plot of the average of annuli around the cell -center against the radial distance from the cell-center, and angular profile (right) plots the average of cell-centered pie slices against polar angle. Black and blue dashed lines indicate the 95% confidence intervals of the shuffled images for excitatory and inhibitory inputs respectively. **(E)** Overlay of RC (left) and ML (right) marginals from inhibitory and excitatory maps demonstrate slightly broader extent of inhibitory inputs in RC direction (arrow).

To identify an eventual underlying spatial pattern of connectivity, we first calculate the probability to observe a connection from a particular relative location in space. For the population of recorded neurons we align neurons at their soma with respect to the cardinal axes (ML and RC, see Methods). After alignment, we plot the probability of obtaining an EPSC or IPSC (>8 ms latency) at each stimulus location, which we refer to as the connection probability. The connection probability simply represents the average percent of responses at a particular location relative to the soma divided by the total number of times this position is photostimulated (typically once per recorded neuron). Both mean excitatory (*N* = 26 cells and *N* = 7 slices) and inhibitory (*N* = 24 cells and *N* = 7 slices) connection probability maps show that inputs can be evoked from a large area (**Figures 4A,B**). The marginal profiles along the cardinal orientations (ML vs. RC; **Figure 4C**) show peaks near the soma with connection probability decreasing with increasing radial distance from cell-center. The extent of the excitatory and inhibitory RC marginal is somewhat broader than that of the ML marginals (**Figure 4C**, arrow). The shape resembles that of a 2D Gaussian, as expected from paired patch recordings (Oswald et al., 2009; Levy and Reyes, 2012). Areas near to the soma show a decrease in connection probability, which is due to the masking of both response types by direct photo-stimulation currents (“doughnut-hole” in the center of maps in **Figures 4A,B**). Moreover the marginal profiles in the RC and ML directions demonstrate patchiness with some local peaks, particularly in the ML direction. To characterize the decrease in connection probability with distance we plot the connection probability in polar coordinates. The mean radial profile shows that there is significantly greater connection probability within 240 μm from cell-center for both excitatory and inhibitory connections (**Figure 4D**, left). The angular profiles showed undulations suggesting spatial periodicities (**Figure 4D**, right). A comparison on the marginal profiles of excitation and inhibition revealed a similar spatial extent with a bias for inhibitory connections to be broader in the RC direction (**Figure 4E**, arrow).

**FIGURE 5:**
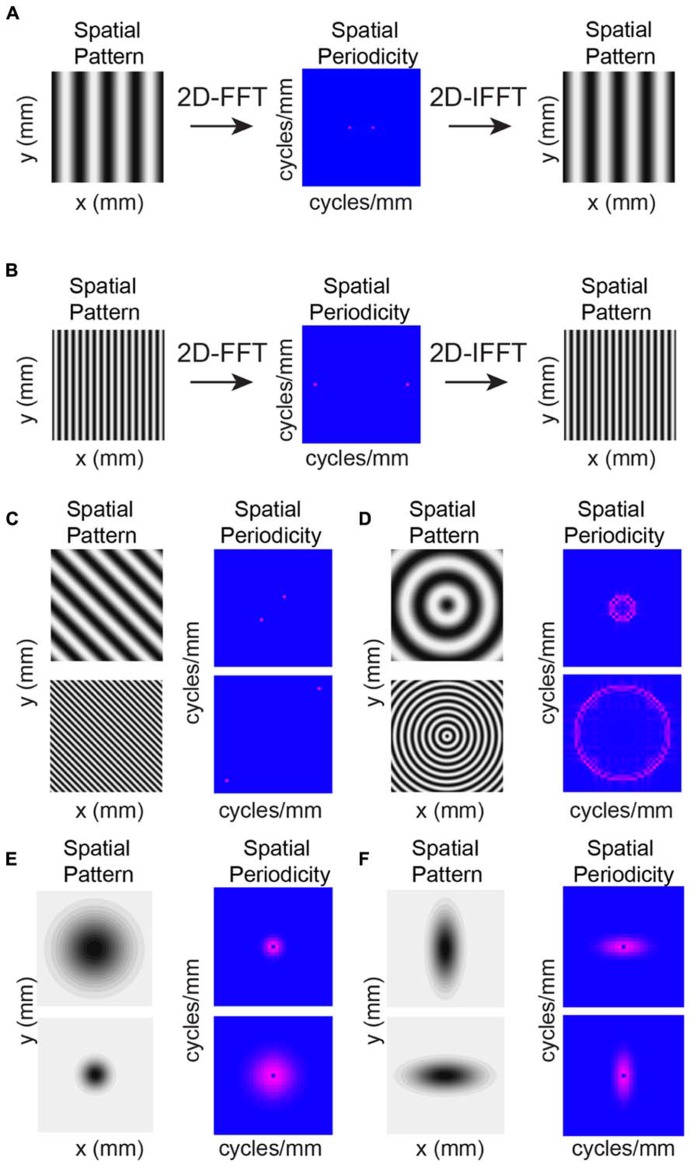
**Example 2D Fourier Analysis (FFT) images demonstrate conversion from space to spatial periodicity.** Analysis applied to connection probability maps demonstrate significant periodicities. **(A,B)** Sample 2D FFTs. Simple oriented gratings result in single points in spatial periodicity with higher frequency gratings resulting in higher frequency spatial components. Applying the inverse 2D FFT (IFFT) recreates the original image with these spatial frequencies. **(C)** Changing the orientation of the grating results in spatial frequency components aligned in the same direction as the grating. **(D)** Periodicity in all directions results in a circular profile in spatial periodicity. **(E)** A 2D Gaussian spatial profile transforms into a 2D Gaussian of spatial periodicity. **(F)** Anisotropies in the Gaussian spatial profiles will lead to anisotropies in the 2D Gaussians periodicity profiles but at orientations orthogonal to the spatial profile.

The individual maps (**Figure 3**) and marginal distributions (**Figures 4C,D**) show peaks, suggesting spatial periodicities. We thus investigate whether, on average, neurons demonstrate significant spatial periodicity of inputs and also if significant periodicities show any spatial bias with respect to RC or ML direction. To reveal spatial periodicities, we perform a two-dimensional (2D) Fourier Analysis (FFT) of connection probability maps. The 2D FFT converts an image into spatial frequency components that essentially represent how the original image can be reconstructed as the linear combination of gratings at different periodicities and in different directions (**Figure 5**). Spatial periodicities will be evident in the FFT maps as significant components in a particular direction (see Methods). If these periodicities are anisotropic, e.g., present in only one direction, then significant FFT components will only be present in one direction. One can convert from significant components at a particular frequency back to the spacing of the peaks in the original map in the same manner as the conversion between time and frequency (for one-dimensional signals). This operation in effect reduces noise in the connection probability map by removing frequency components that are not significant. The significant components in the 2D FFT define the spatial spacing of peaks in the original maps. For example, a significant component in the 2D FFT at 10 mm^-^^1^ represents peaks in the original image at a spacing of 100 μm while a significant component at 5 mm^-^^1^ represents spatial peaks at 200 μm. The spatial periodicity of a grating in a particular direction is simply a single point (two points because of Fourier component symmetry) in the direction of the grating relative to the origin (**Figures 5A–C**). Thus a grating occurring in one dimension (e.g., along the *x*-axis) will be represented by two distinct spatial frequency components long the *x*-axis (**Figures 5A,B**). A diagonal grating in space would have spatial frequency components in both the *x*- and *y*-axes (**Figure 5C**). A spatial sinusoid in all directions becomes a circle in spatial periodicity with the radius of the circle being larger for higher spatial periodicities (**Figure 5D**, compare upper and lower). A 2D Gaussian spatial profile transforms into spatial periodicity components that also appear as a 2D Gaussian in spatial frequency space (**Figure 5E**). Importantly, a Gaussian that is wider in the spatial domain is narrower in spatial frequency domain and vice versa. Thus, an anisotropic 2D Gaussian in space is also anisotropic in frequency but with the wider direction in space being narrower in frequency and vice versa (**Figure 5F** compare upper and lower).

**FIGURE 6:**
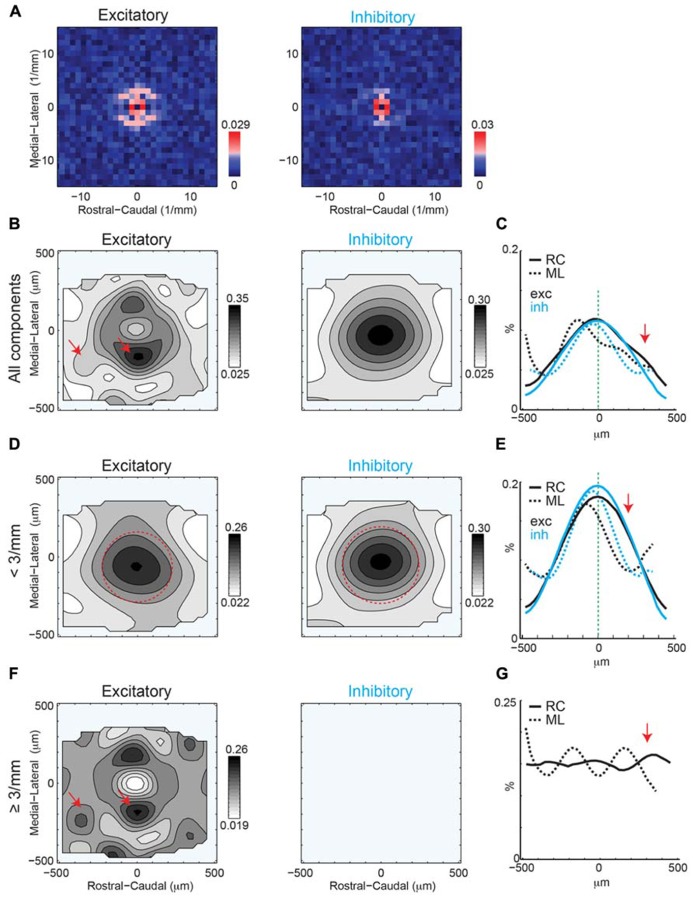
**(A)** 2D Fourier transforms of the mean connection probability maps demonstrate anisotropy of significant spatial frequency components along the ML (isofrequency) for excitatory inputs but relative symmetry of significant spatial frequency for inhibitory inputs. Red pixels indicate significant components (*p* < 0.05, bootstrap test). **(B)** Reconstruction of spatial maps using only the significant frequency components (red pixels in **A**). Red arrows show anisotropic patches at different orientations and distances. **(C)** Marginals in the RC and ML direction. Note that RC marginal are wider than ML marginal (arrow). Full width at half max for excitation: ML 326 μm vs. RC 505 μm; for inhibition: ML 288 μm vs. RC 500 μm. **(D,E)** Reconstruction and marginals of only the low spatial frequency (<3 mm^-^^1^) components. Red circle in D is plotted in comparison to highlight the anisotropy of the spatial pattern between ML and RC directions. The arrow in **E** highlights the anisotropy in the RC direction. Full width at half max for excitation: ML 282 μm vs. RC 516 μm; for inhibition: ML 288 μm vs. RC 510 μm. **(F,G)** Reconstruction and marginals of only the high spatial frequency (≥3 mm^-^^1^) components. Red arrows highlight the anisotropic patches in the excitatory map.

2D Fast Fourier Transform analysis of excitatory and inhibitory inputs reveals significant components at multiple spatial frequencies (**Figure 6A**). The central significant components (with the lowest spatial frequency) form a ring, reflecting the central 2D Gaussian distribution of the connection profile (see **Figure 4D**). 2D FFT analysis of excitatory inputs reveals multiple significant components at higher spatial frequencies (~300 μm), with more components and “hot spots” in the ML directions (**Figure 6A**). Thus, on average excitatory inputs have anisotropic spatial frequency components, with periodic hot spots of connectivity biased to the ML direction. However, the spatial periodicity of inhibition does not show this same number of high spatial frequency components and only a weak anisotropy of spatial periodicity (**Figure 6A**). Significant spatial frequency components for inhibition were clustered at low frequencies, indicative of a wide 2D Gaussian, as apparent from the mean connection probability map (**Figure 4B**). However, the significant components of inhibitory inputs are somewhat more spread in the ML direction consistent with a somewhat wider profile in the RC direction (see **Figure 4C**).

To visualize the spatial patterns reflected in the significant components we perform an inverse 2D FFT (I2DFFT) on the significant components. This reconstruction thus highlights portions of the spatial structure that are significant based on the bootstrap test (*p* < 0.05). The reconstructed excitatory connection probability map shows a “doughnut” shape with two areas of high connection probability ~200 μm from the soma in the ML direction (**Figure 6B**, left, red arrows). The reconstructed inhibitory probability map shows a 2D Gaussian profile (**Figure 6B**, right). To compare more closely the spatial shape of the reconstructed maps we plot the marginal profiles along the RC and ML direction (**Figure 6C**). We find that both excitatory and inhibitory profiles are wider (full width at half max excitation: ML 326 μm vs. RC 505 μm; inhibition: ML 288 μm vs. RC 500 μm) in the RC direction than the ML direction indicating an anisotropy in the spatial pattern. This spatial anisotropy is consistent with the anisotropy of the significant spatial frequency components (see **Figures 6A** and **5D**). While the inhibitory marginal shows a Gaussian shape, the excitatory marginal shows a widening in the caudal direction. To investigate this further we separate the central spatial components (<3 mm^-^^1^ spatial frequency), which have large amplitudes and thus dominate the profile. Plotting the connection probability map for the central components shows the spatial profile of the central (<3 mm^-^^1^) 2D Gaussian (**Figure 6D**). Both the excitatory and inhibitory profiles are elongated in the RC direction (red circles), which is also evident from the broader marginal profiles in the RC direction than the ML direction (**Figure 6E**; full width at half max excitation: ML 282 μm vs. RC 516 μm; inhibition: ML 288 μm vs. RC 510 μm). We then re-plot the connection probability maps for the higher spatial frequency components. The excitatory map shows the large peaks in the ML direction visible before, but also additional smaller amplitude peaks ~350 μm in RC from the soma (**Figure 6F**, arrows). In contrast after removal of the central components, the inhibitory connection probability map does not show any additional peaks. The marginal profiles also show the spatial periodicity with a period of ~350 μm in RC and ~300 μm in ML (**Figure 6G**). Together these analyses show that the intralaminar connection probability in layer 2/3 of A1 is spatially anisotropic and also shows spatially anisotropic connectivity peaks visible as periodicities in the 2DFFT. These periodicities have a spatial frequency of ~300–350 μm.

### INTRALAMINAR CONNECTION STRENGTHS IN LAYER 2/3 OF A1 SHOW ANISOTROPIC PERIODICITIES

**FIGURE 7:**
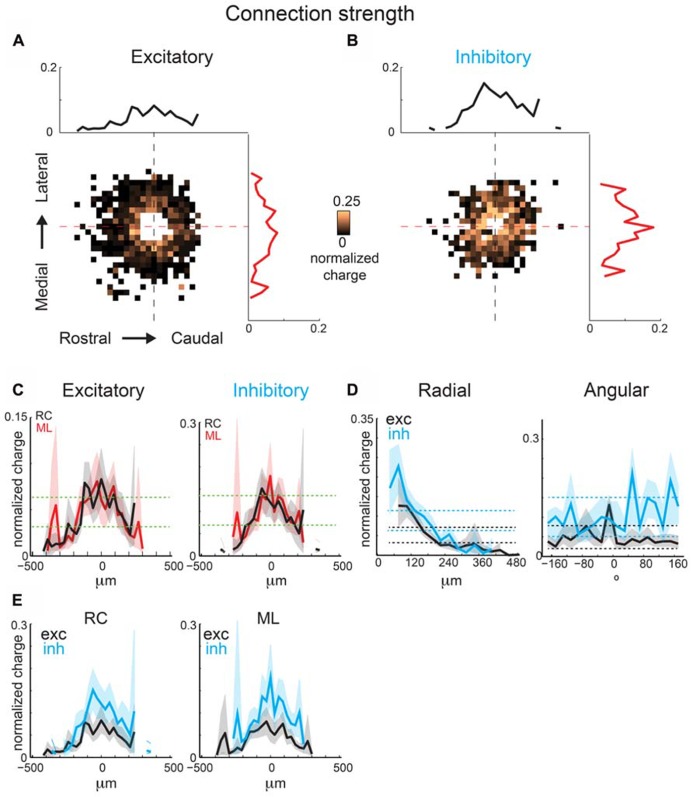
**Mean strength maps from the same population of neurons as in Figure 4 are consistent with connection probability trends.** Excitatory **(A)** and inhibitory **(B)** mean strength maps show increased patchiness relative to mean connection probabilities. **(C)** Overlaid ML and RC marginal profiles from **(A,B)** for excitatory (left) and inhibitory (right) inputs. Green dashed lines indicate the 95% confidence interval for the mean of the shuffled image. The shaded areas for this and subsequent plots represent the 95% confidence interval for the marginal profiles. **(D)** Overlaid polar marginal profiles of the mean strength maps. Black and blue dashed lines indicate the 95% confidence intervals of the shuffled images for excitatory and inhibitory inputs respectively. **(E)** Overlaid ML and RC marginal profiles from C comparing inhibition and excitation. Note that inhibition shows more asymmetry in RC and ML direction than excitation.

The above analysis focuses on connection probability. However, inputs originating from certain spatial locations might have different synaptic strengths. We investigate this hypothesis by calculating the spatial distribution of event amplitudes, measured as average normalized total charge (normalized per single map), which we refer to as mean strength maps. Connection probability is the total number of times an event occurred at a photostimulation location divided by the total number of times that location was photostimulated over all recorded neurons, whereas mean strength is the mean transferred charge for events that occurred at a photostimulation location. These maps (**Figures 7A,B**) are sparser (since average amplitudes can only be computed where significant connection probability exists) but also patchy, indicating that connections from certain relative positions are particularly strong (for example ~100 μm in RC direction). The marginal distributions are largely overlapping, indicating little spatial anisotropy in connection strength (**Figure 7C**). Excitatory and inhibitory connection strength decrease as a function of radial distance (**Figures 7C,D**). The angular maps show peaks, consistent with the hot spots in the connection maps (**Figures 7A,B**). The inhibitory and excitatory peaks are present at different angles. Together this suggests that excitatory input strength decreases uniformly with distance but hot spots occur at some angular orientations. When compared, inhibition shows more asymmetry in the RC and ML directions than excitation (**Figure 7E**).

**FIGURE 8:**
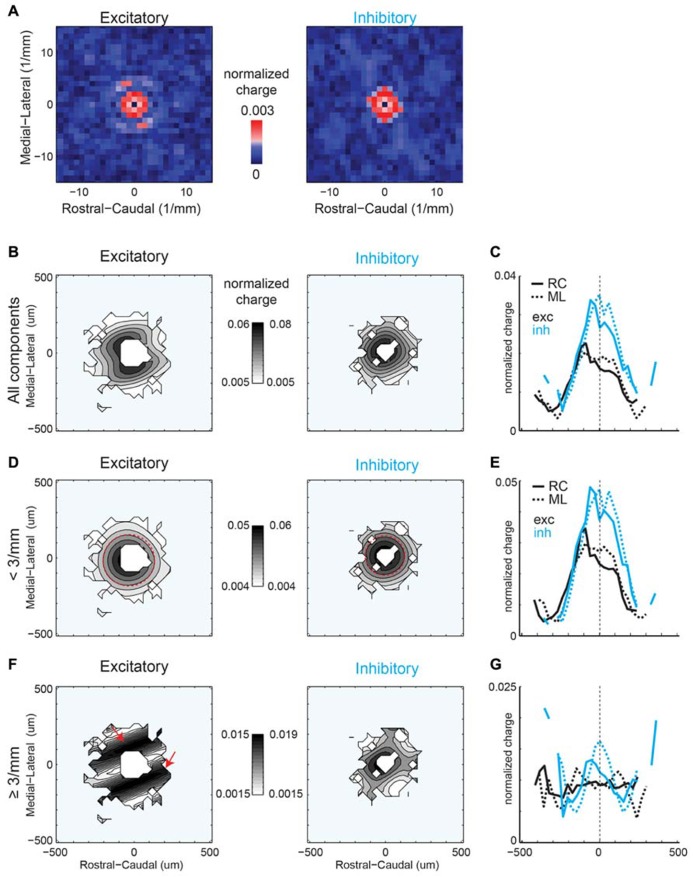
**(A)** Spatial frequency anisotropy for mean strength demonstrates significant patchiness in the ML (isofrequency) direction for excitatory inputs, but symmetry amongst the significant frequency components for inhibitory inputs. **(B)** Reconstruction of spatial maps using only the significant frequency components (red pixels in **A**). **(C)** Marginals in the RC and ML direction. **(D,E)** Reconstruction and marginals of only the low spatial frequency (<3 mm^-^^1^) components. Red circle in **D** is plotted in comparison to highlight the symmetry of the spatial pattern between ML and RC directions. **(F,G)** Reconstruction and marginals of only the high spatial frequency (≥3 mm^-1^) components. Red arrows highlight the anisotropic bands in the excitatory map.

Spatial periodicity analysis of mean strength maps shows significant high spatial frequency components for excitatory inputs offset in the ML and RC direction, indicating anisotropy (**Figure 8A**), consistent with the connection probability maps (**Figure 6A**). The reconstructed map of excitatory mean strength (**Figure 8B**) and marginal distributions (**Figure 8C**) shows the overall isotropic low spatial frequency decrease in strength (**Figures 8D,E**, red circles). This is in contrast to the anisotropy observed in spatial connection probability (**Figure 6C**). Moreover, the reconstructed map of excitatory mean strength (**Figure 8B**) and marginal distributions (**Figure 8C**) also shows that the areas of high mean strength form diagonal lobes. This feature becomes even more evident when the central components are removed (**Figure 8F**, red arrows, **8G)**. Thus excitatory inputs originating from a band angled ~30 degrees in the ML directions are of higher strength. Inhibitory spatial periodicities show high frequency components, but without spatial bias (**Figures 8A,B,D**). While the lack of spatial bias was consistent with the dominant 2D Gaussian pattern also seen in the connection probability (**Figure 6A**), the largest components were not closest to the soma but where of higher frequency. This is likely due to the fact that inhibitory inputs close to the soma are reduced because of shunting due to direct activation of the neuron. When the central components are subtracted ~150 μm wide bands of high mean strength become visible in the RC and ML orientations.

### DENDRITIC ORIENTATION IN AUDITORY AND VISUAL CORTICES DOES NOT SHOW SPATIAL ANISOTROPY

**FIGURE 9:**
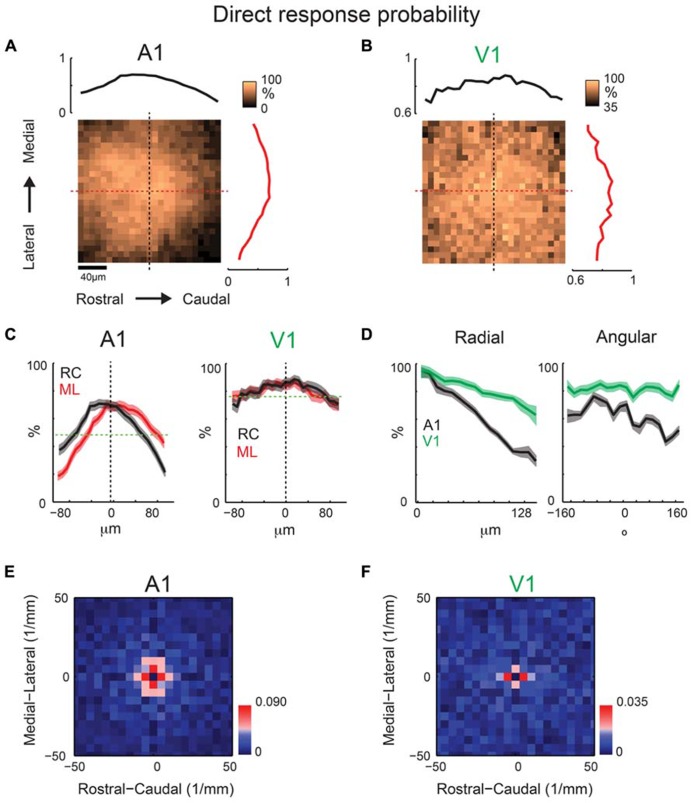
**Mean direct probability maps from a population of neurons recorded at 40× reveal spatially periodic dendritic structure.** Mean maps from a population of A1 (**A**; *N* = 33) and V1 (**B**; *N* = 20) recorded neurons demonstrating relatively symmetric profile of basal dendrites in both A1 and V1. Plots above and to the right of the mean map indicate the marginal profile along the ML (isofrequency) and RC (tonotopic) directions, respectively. **(C)** Overlaid ML and RC marginals from **(A,B)** for A1 (left) and V1 (right). Green dashed line indicates the mean of the shuffled image and the black dashed line indicates cell-center. The shaded areas for this and subsequent plots represent the 95% confidence interval for the marginal profiles. **(D)** Mean map polar marginals where the radial profile (left) is a plot of the average of annuli around the cell-center against radial distance from the cell-center, and the angular profile (right) plots the average of cell-centered pie slices against the polar angle. Polar marginals from A1 and V1 are overlaid to demonstrate the wider extent of the average basal dendritic arbor in V1 relative to A1. **(E,F)** 2D Fourier transforms of the mean direct probability maps demonstrate relatively symmetric significant components in A1 **(E)** and V1 **(F)**. Red pixels indicate significant components (*p* < 0.05, bootstrap test).

Our results show periodic regions of higher connection probability along the isofrequency and tonotopic direction. To investigate if this anisotropy of spatial periodicity is reflected by the underlying dendritic architecture, we investigate if proximal dendritic trees showed significant spatial or spatial periodicity patterning. We use the spatial pattern of the direct response recorded while photostimulating at 40× magnification in order to characterize the shape of the proximal dendritic tree. Evoked excitatory responses close to the soma or proximal dendrites are masked by the direct response (see **Figure 2**) allowing us to measure the spatial extent of the proximal dendritic tree. We create average maps analogous to connection probability, but instead of connection probability these maps indicate the probability of a direct response at each location relative to the soma. The resulting maps (**Figures 9A,B**) and marginal profiles (**Figure 9C**) reveal a roughly symmetric pattern, thus indicating that the basal dendrites of layer 2/3 neurons in A1 (*N* = 33) show no spatial anisotropy. The radial profile demonstrates decreased probability as a function of distance, consistent with decreased dendritic density and attenuation of the direct dendritic response (**Figure 9D**). The angular profile is consistent with the rostral-lateral bias from the RC and ML profiles (**Figure 9D**). We also perform the spatial periodicity analysis with the direct probability maps. We find that the significant components of the 2D FFT are also spatially symmetric (**Figure 9E**). The spatial periodicity shows significant components (*p* < 0.05, red pixels) in all directions, also visible in the angular profile (**Figure 9D**). Together our data indicate that on average there is no spatial or spatial periodicity anisotropies of dendritic orientation in A1.

In order to test whether the absence of a spatial arrangement of basal dendritic arbors was specific to auditory cortex or was a general cortical feature, we also record from layer 2/3 neurons in tangential slices of primary visual cortex (V1). These slices are created by cutting perpendicularly to the thalamocortical V1 slice preparation (MacLean et al., 2006). Average direct response probability maps (*N* = 20 neurons) demonstrate a similar result as those in A1, with a roughly spatially symmetric pattern (**Figures 9B–D**). Similar to A1, V1 marginal profiles show a symmetric periodic structure (**Figure 9F**). The spatial frequency components in V1 are smaller than those in A1 with significant components being 200 μm in V1 and 100–200 μm in A1, implying a wider spatial extent of the basal dendritic tree in V1 vs. A1.

### LOCAL A1 BUT NOT V1 INHIBITION IS PERIODIC IN ML (ISOFREQUENCY) DIRECTION

**FIGURE 10:**
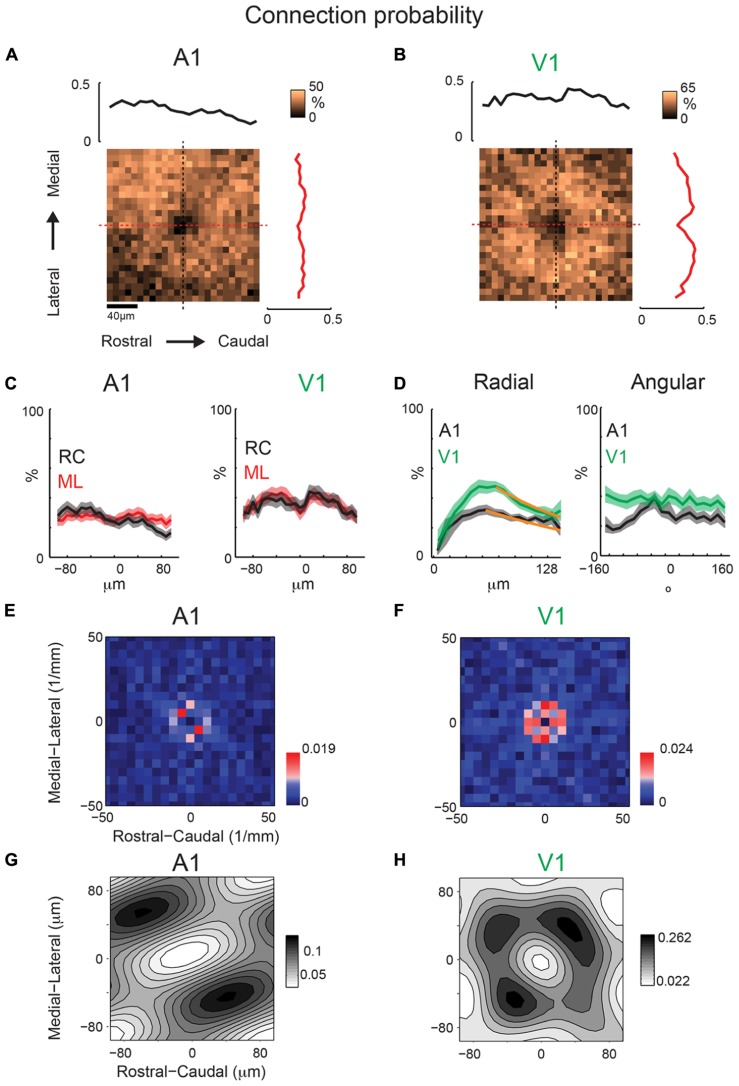
**Mean inhibitory connection probability maps from a population of neurons recorded at 40 × show spatial symmetry and anisotropic spatial periodicity in ML (isofrequency) direction.** Mean maps from a population of A1 (**A**; *N* = 31) and V1 (**B**; *N* = 20) recorded neurons demonstrating relatively symmetric profile of inhibitory inputs in both A1 and V1. Plots above and to the right of the mean map indicate the marginal profile along the ML (isofrequency) and RC (tonotopic) directions, respectively. **(C)** ML and RC marginals from **(A,B)** overlaid for A1 (left) and V1 (right). **(D)** Mean map polar marginals where the radial profile (left) is a plot of the average of annuli around the cell-center against radial distance from the cell-center, and the angular profile (right) plots the average of cell-centered pie slices against the polar angle. Overlaid polar marginals from A1 and V1 show the steeper radial decay of connection probability in V1 vs. A1 (spatial exponential decay constants of λ = 112 μm and λ = 207 μm, respectively). Single exponential decay fits are shown as orange lines. **(E,F)** 2D Fourier transforms of the mean inhibitory connection probability maps demonstrate spatial frequency anisotropy is present in A1 **(E)** in the ML (isofrequency) direction, but absent in V1 **(F)**. Red pixels indicate significant components (*p* < 0.05, bootstrap test). **(G,H)** show the reconstructions of the inhibitory connection probability maps from the significant frequency components. Note diagonally oriented hot spots in A1 while hot spots are isotropically arranged in V1.

We next investigate whether inhibitory maps in A1 and V1 have similar or different spatial anisotropies. While inhibitory maps recorded near the soma of individual neurons are patchy (**Figures 2B,C**), inhibitory connection probability maps (at 40× magnification, 200 × 200 μm) from A1 (*N* = 31 cells, *N* = 14 slices) and V1 (*N* = 20 cells, *N* = 14 slices) are roughly spatially symmetric (**Figures 10A,B**). Marginal profiles of A1 mean inhibitory connection probability maps demonstrate a slight bias toward the lateral direction (**Figure 10C**). This bias is not present in the RC direction. Outside of the area of direct activation where inhibitory currents are potentially masked by direct currents, the radial profile demonstrates a decrease in connection probability with distance (**Figure 10D**), consistent with previous studies (Oswald et al., 2009). Marginal profiles of V1 inhibitory maps also demonstrate symmetry in the RC and ML directions (**Figure 10C**). We fit the radial profiles (**Figure 10D**) with a single exponential decay function after the peak and the decay constant for A1 (λ = 207 μm) is about twice as long as that for V1 (λ = 112 μm). Thus the radial falloff of connection probability is much wider for A1 compared to V1, indicating a wider extent of inhibitory inputs in A1.

We next perform the spatial periodicity analysis to test if, on average, local inhibitory inputs demonstrated significant repeating areas of high input probability. The 2D FFT of the A1 mean inhibitory map shows significant components (*p* < 0.05) along the ML (isofrequency) direction (**Figure 10E**). This suggests that locally A1 layer 2/3 neurons receive functional inhibitory synaptic inputs from locations that are spatially non-uniform, with a periodic structure of high inhibitory connection probability in the isofrequency direction. In contrast to A1 mean maps, V1 mean maps do not show anisotropy of significant spatial periodicity (**Figure 10F**). Together these results show a distinct difference in the spatial distribution of inhibitory connections in A1 and V1 (**Figures 10G,H**).

**FIGURE 11:**
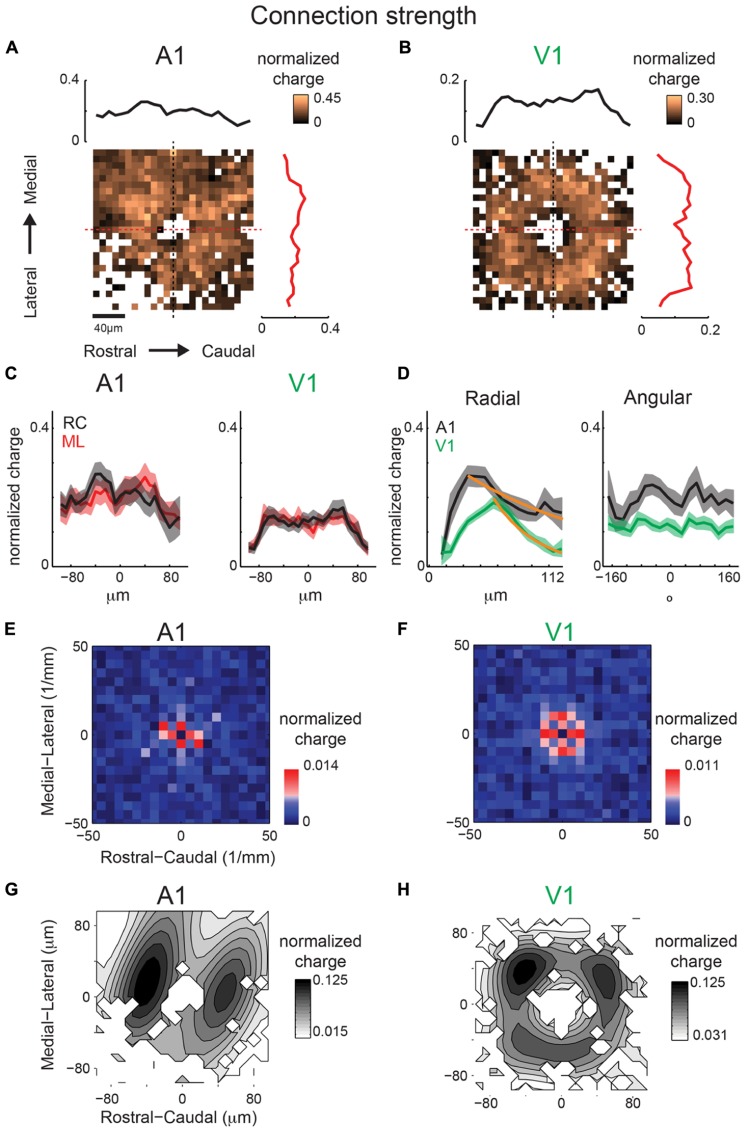
**Mean inhibitory IPSC strength maps from the same population of neurons as in Figure 10 show consistent trend with connection probability.** Mean maps from the population of A1 **(A)** and V1 **(B)** recorded neurons demonstrating profile of strength of inhibitory inputs in both A1 and V1. Plots above and to the right of the mean map indicate the marginal profile along the ML (isofrequency) and RC (tonotopic) directions respectively. **(C)** ML and RC marginals from **(A,B)** overlaid for A1 (left) and V1 (right). Radial dropoff of mean strength **(D)** shows the same trend as mean connection probability – a steeper decay in V1 relative to A1 (spatial exponential decay constants of λ = 43 μm and λ = 138 μm, respectively), although the peak for inhibition is further from the cell-center for V1. Single exponential decay fits are shown as orange lines. Black and blue dashed lines indicate the mean of the shuffled images for A1 and V1 inhibitory inputs, respectively. **(E)** Spatial frequency anisotropy in A1 for mean strength demonstrates significant patchiness in the RC (tonotopic) direction, the opposite direction as the anisotropy in A1 connection probability maps. Significant components shown in red (*p* < 0.05, bootstrap test). **(F)** Spatial frequency for V1 is relatively symmetric. **(G,H)** show the reconstructions of the mean IPSC strength maps from the significant frequency components. Note anisotropic hot spots in A1 while hot spots are isotropically arranged in V1.

Similar to our analysis of maps obtained under 10× magnification, we also calculate inhibitory mean strength maps recorded at 40×. A1 and V1 maps are relatively symmetric in space (**Figures 11A,B**). V1 maps of connection strength show the same degree of symmetry as for connection probability in the RC and ML marginal profiles (**Figure 11C**). Radial marginal profiles indicate a significant peak at 104 μm from cell-center for A1 but not for V1 (**Figure 11D**). Although the peak of inhibitory mean strength in the radial profile is further out than for connection probability, the spatial decay from this point out is still steeper for V1 (λ = 43 μm) than for A1 (λ = 148 μm), consistent with the radial profiles of connection probability (**Figure 10D**). We also calculate 2D FFT of the mean connection strength maps. Increased patchiness of the mean strength relative to connection probabilities for A1 is reflected in significant components in the RC direction (**Figure 11E**). Mean strength maps for V1 are symmetric in spatial frequency (**Figure 11F**). Thus overall A1 is patchier than V1 in connection probability and in connection strength (**Figures 10G,H** and **11G,H**). Yet the anisotropy of connectivity in A1 is more ML for mean connection probability and more RC for mean strength. Part of this may have been due to a larger masking effect on inhibitory inputs by direct responses (compare sizes of “doughnut hole” in **Figures 10A** and **11A**), making the connection probability a more reliable metric of overall anisotropy than mean strength.

## DISCUSSION

**FIGURE 12:**
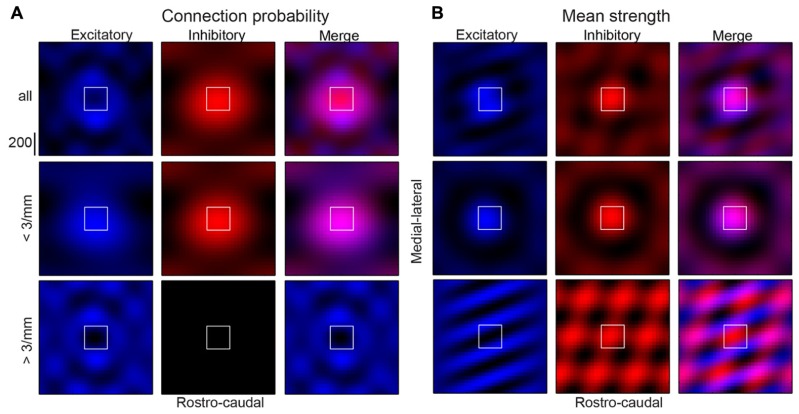
**Summary of connection probability (A) and mean strength (B) results demonstrating “average” A1 layer 2/3 neuron (top rows).** Inhibitory inputs are in red (left columns), excitatory inputs are in blue (center columns) and both are shown overlaid (right columns). Images were created with inverse 2D FFT of only the significant components for the corresponding maps (pixels in red in connection probability 2D FFT images) meaning they only reflect significant spatial structure from the original maps. Periodicities that comprise the central “bump” only (middle rows) are highlighted by reconstructing with only the significant low frequency components, while those beyond the central “bump” (bottom rows) are highlighted by reconstructing without the lowest frequency components.

We show that a patchy, anisotropic, and periodic connectivity pattern for inhibitory and excitatory inputs exists in layers 2/3 of auditory cortex. While excitatory and inhibitory connection probabilities are broader in the tonotopic axis (**Figures 6C,E**), their significant components of spatial periodicity are aligned perpendicular to the tonotopic axis (isofrequency direction). Similar experiments in visual cortex did not reveal a spatial bias of periodicity for inhibitory inputs at smaller spatial scales. Excitatory connection strength is relatively spatially uniform, but demonstrates a spatial periodicity with patchiness diagonally but primarily in the direction of the isofrequency axis. Results are summarized in **Figure 12** with a schematic representation of the connectivity to an “average” A1 layer 2/3 neuron based on the low magnification findings. The summary figure was created by taking on the significant components from the spatial periodicity analysis (**Figures 6** and **8**; red pixels), recreating the connection probability and mean strength images with only these components, and overlaying excitatory and inhibitory maps (**Figure 12**, right columns). The low-frequency periodicities comprise the central “bump” (**Figures 12A,B**, middle row). By omitting low frequencies, these plots highlight the significant higher frequency components (**Figures 12A,B**, bottom row), showing connectivity “hot-spots” further away from the soma. The reconstructions demonstrate the relative symmetry of periodicity for inhibitory inputs, but spatial periodicity being predominantly in the ML direction for excitatory inputs (lower left panels of **Figures 12A,B**).

Since prior *in vitro* studies of A1 have found tonotopically aligned asymmetries and unique connectivity of inter-laminar connections in A1 (Barbour and Callaway, 2008; Oviedo et al., 2010), similar spatial biases might also exist in intra-laminar connections or in intra-laminar connection within deeper layers, for example layers 5/6 of A1. Because we have found spatial and spatial-frequency anisotropies that appear to be unique to A1 (or at least not found in V1 inhibitory connections), our results show that A1 contains unique circuits that shape its responses and that the cerebral cortex is not a uniform processor, but that different regions contain microcircuits that are specialized to a particular processing task and with the different spatial layout of the sensory end organs, such as a 1-dimensional cochlea vs. a 2-dimensional retina.

In particular we find that despite on average what is likely a larger extent of proximal dendrites in V1 relative to A1 (**Figure 10**), the radial decay of inhibitory connection probability and inhibitory mean strength is steeper in V1 than in A1 (**Figures 11** and **12**). This indicates a more important role of inhibition and the radius of strong inhibitory inputs in A1. This could likely be the substrate for either co-tuned response shaping, i.e., for sound level response shaping or temporal response modulation (Wehr and Zador, 2003; Wu et al., 2006; Tan et al., 2007), or reflect inhibitory inputs’ role in lateral inhibition (Wang et al., 2002; Wu et al., 2008). It has been suggested that thalamic inputs could potentially recruit different areas of inhibition (de la Rocha et al., 2008; Levy and Reyes, 2011) in a manner that can potentially generate the variety of level and frequency tuning properties that are often reported in A1 neurons.

Our findings here are likely understated in terms of the degree of anisotropy in A1 vs. V1 for several reasons. (1) The orientation of the tonotopic maps relative to anatomical landmarks in mice is known to be highly variable (Stiebler et al., 1997), because our slices were always aligned using anatomical landmarks, if connectivity patterns of neurons are indeed aligned to the tonotopic or other physiological topographies, then variability between animals can easily lead to a blurring of this observed connectivity structure. (2) It is difficult for us to record true EPSCs or IPSCs near to the soma due to direct photoactivation of the neuron (see **Figure 2**). This may obscure any trends that could be seen within approximately 100 μm of the soma. (3) Our tangential slice preparation removes the apical dendrite and any dendritic or axonal branching patterns and connectivity within layer 1. Thus, our findings here primarily represent basal dendrite connectivity of layer 2/3 cells in A1. (4) Because of the larger point-spread function, single-photon LSPS is quite likely to be activating more than one neuron (but likely fewer than 50 neurons Shepherd et al., 2005). This means any underlying fine spatial patterns could be obfuscated by multiple neuron connectivity patterns being measured simultaneously at one photostimulation location. (5) Due to our tangential slice technique, it is difficult for us to know where a recorded neuron is located within layer 2/3, meaning some neurons could be layer 3 neurons and others mostly layer 2 neurons. If any difference in connectivity patterns exists between these layers, we would not be able to tease these apart with our current dataset.

Some previous studies of connectivity in auditory cortex have employed paired recordings. This allows the experimenter to know the cell identity of both neurons based on physiological response properties and *post hoc* histology. Although LSPS allows identification of the patched (postsynaptic) cell, with the LSPS technique it is difficult to know which presynaptic neuron or neurons are being photoactiviated. However, the LSPS technique allows a much larger number of presynaptic areas to be sampled, so is able to cover a much larger radial distance from each postsynaptic neuron from which PSCs are being recorded. Moreover by using FFT analysis we are able to detect spatial connectivity patterns that would not be detectable using sparse sampling via paired recordings

The periodicity of excitatory and inhibitory inputs in the isofrequency direction suggests that activity in local circuits that may have similar spectral properties could inhibit each other. This could serve as a mechanism for a circuit to inhibit potentially competing circuits that have similar superthreshold frequency responses but that specialize in processing features that are not behaviorally relevant to the current environment. For example, if the current task is discriminating narrowband sounds, two circuits that are contained in a specific isofrequency band, one that integrates over a large bandwidth and another that only responds to narrowband sounds, periodic inhibitory connections might allow the narrowband circuit to inhibit the broadband circuit because of its greater behavioral relevance (Read et al., 2001). The observed periodicity along the tonotopic axis at ~300 μm could allow neurons to selectively integrate distinct frequency bands and lead to multipeaked tuning curves (Winkowski and Kanold, 2013). Given that in mouse best frequency varies at ~3 oct/mm (Bandyopadhyay et al., 2010), such connection width could indicate an underlying octave spacing of intralaminar connections in layer 2/3. Moreover, the wide area of inputs across the tonotopic axis (~600 μm) indicates that layer 2/3 neurons can sample a large frequency space consistent with recent spine imaging studies (Chen et al., 2011). On a larger spatial scale, functional roles for periodicity of connections along and across isofrequency bands have previously been suggested. These include connections amongst binaural regions (Reale et al., 1983), regions of equal or greater CF (Matsubara and Phillips, 1988), regions of differing CF for spectral integration (Ojima and Takayanagi, 2004), clustered regions along isofrequency bands (Ojima et al., 2005) and regions with similar spectral bandwidth (Read et al., 2001).

Together our results show the existence of a specific microstructure of connections within auditory cortex and suggest that the heterogeneous organization of auditory cortex might be assembled using spatially precise interleaved microcircuits.

## Conflict of Interest Statement

The authors declare that the research was conducted in the absence of any commercial or financial relationships that could be construed as a potential conflict of interest.
